# Impact of prior antibiotic therapy on severe necrotizing soft-tissue infections in ICU patients: results from a French retrospective and observational study

**DOI:** 10.1007/s10096-021-04354-8

**Published:** 2021-10-09

**Authors:** Sébastien Tanaka, Michael Thy, Parvine Tashk, Lara Ribeiro, Brice Lortat-Jacob, Jean-François Hermieu, Nathalie Zappella, Sacha Rozencwajg, Aurelie Snauwaert, Enora Atchade, Nathalie Grall, Maksud Assadi, Alexy Tran-Dinh, Philippe Montravers

**Affiliations:** 1 Assistance Publique - Hôpitaux de Paris (AP-HP), Department of Anesthesiology and Critical Care Medicine, DMU PARABOL, Bichat-Claude Bernard Hospital, 46 rue Henri Huchard, 75018 Paris, France; 2 French Institute of Health and Medical Research (INSERM), U1188 Diabetes Atherothrombosis Réunion Indian Ocean (DéTROI), CYROI Plateform, Réunion Island University, Saint-Denis de La Réunion, France; 3 Université de Paris, Paris, France; 4 EA 7323 - Pharmacology and Therapeutic Evaluation in Children and Pregnant Women, Paris Descartes University, Sorbonne Paris Cité University, Paris, France; 5 Assistance Publique - Hôpitaux de Paris (AP-HP), Department of General and Visceral Surgery, Bichat-Claude Bernard Hospital, Paris, France; 6 Assistance Publique - Hôpitaux de Paris (AP-HP), Department of Urology, Bichat-Claude Bernard Hospital, Paris, France; 7 Assistance Publique - Hôpitaux de Paris (AP-HP), Department of Bacteriology, Bichat-Claude Bernard Hospital, Paris, France; 8 French Institute of Health and Medical Research (INSERM), IAME, UMR 1137, Paris, France; 9 Laboratory for Vascular Translational Science, French Institute of Health and Medical Research (INSERM) U1148, Paris, France; 10 Physiopathology and Epidemiology of Respiratory Diseases, French Institute of Health and Medical Research (INSERM) U1152, ANR10-LABX-17, Paris, France

**Keywords:** Necrotizing soft-tissue infection, Sepsis, Intensive care unit, Antimicrobial therapy, Antibiotic exposure, Multidrug-resistant infection

## Abstract

Necrotizing soft-tissue infection (NSTI) is a life-threatening pathology that often requires management in intensive care unit (ICU). Therapies consist of early diagnosis, adequate surgical source control, and antimicrobial therapy. Whereas guidelines underline the need for appropriate routine microbiological cultures before starting antimicrobial therapy in patients with suspected sepsis or septic shock, delaying adequate therapy also strongly increases mortality. The aim of the present study was to compare the characteristics and outcomes of patients hospitalized in ICU for NSTI according to their antimicrobial therapy exposure > 24 h before surgery (called the exposed group) or not (called the unexposed group) before surgical microbiological sampling. We retrospectively included 100 consecutive patients admitted for severe NSTI. The exposed group consisted of 23(23%) patients, while 77(77%) patients belonged to the unexposed group. The demographic and underlying disease conditions were similar between the two groups. Microbiological cultures of surgical samples were positive in 84 patients and negative in 16 patients, including 3/23 (13%) patients and 13/77 (17%) patients in the exposed and unexposed groups, respectively (*p* = 0.70). The distribution of microorganisms was comparable between the two groups. The main antimicrobial regimens for empiric therapy were also similar, and the proportions of adequacy were comparable (*n* = 60 (84.5%) in the unexposed group vs. *n* = 19 (86.4%) in the exposed group, *p* = 0.482). ICU and hospital lengths of stay and mortality rates were similar between the two groups. In conclusion, in a population of severe ICU NSTI patients, antibiotic exposure before sampling did not impact either culture sample positivity or microbiological findings.

## Introduction

Necrotizing soft-tissue infections (NSTIs) often require management in the intensive care unit (ICU). The cornerstone treatment of this entity is based on early diagnosis, adequate surgical source control, and appropriate antimicrobial therapy [1, 2]. Despite these management rules, morbi-mortality is still high [3, 4].

The Surviving Sepsis Campaign highlights the need for appropriate routine microbiological cultures before starting antimicrobial therapy in patients with suspected sepsis or septic shock [5]. Obtaining cultures prior to antimicrobial therapy is usually associated with improved outcomes [6]. However, the need for these microbiological samples must be clearly balanced with the mortality risk of delaying adequate therapy in critically ill patients [7, 8]. In addition, previous antimicrobial therapy before surgical sampling is quite frequent, as reported in the REACH trial, a large European cohort of patients hospitalized for complicated skin and soft tissue infections [9]. In this study, Garau et al. reported that 35.1% of the patients received antibiotics before the initial visit. Interestingly, the consequences of these previous antibiotic regimens are rarely reported, neither in terms of their influence on the results of microbiological cultures nor in the changes in the susceptibility patterns with a potential risk of emergence of multidrug-resistant bacteria [10].

The primary goal of the present study was to investigate the effects of previous antimicrobial therapy on the microbiology of surgical samples in a cohort of severe NSTI ICU patients. The secondary objectives were to describe the microbial ecology in the groups previously exposed vs. unexposed to and to know if starting antimicrobial therapy before surgical management can improve the outcome.

## Material and methods

### Study population

This retrospective study assessed all consecutive patients admitted to our University Hospital ICU for the management of NSTI from April 2009 to March 2019. The identification of the cases was made through the database of the health information system of our hospital using the ICD-10 scores.

This study was declared to the French Data Protection Authority (CNIL: 2096382v0) and was approved by the French Institutional Review Board (Comité d’Éthique de la Recherche en Anesthésie-Réanimation, IRB number 00010254–2020-153). Due to the retrospective nature of our study, the need for signed informed consent was waived.

### Data collection and outcome

Demographic data and severity scores (simplified acute physiology score-II (SAPS-II) and sequential organ failure assessment (SOFA)) were recorded on ICU admission [11, 12]. Septic shock was defined according to SEPSIS-3 definition [5].

The severity of the underlying medical condition (malignancy, obesity, diabetes mellitus, vascular disease, alcohol use, active smoking, and immunosuppression (immune deficiency, HIV, or chronic corticosteroid medication)) was also assessed. The Charlson comorbidity index and the Laboratory Risk Indicators for Necrotizing Fasciitis score (LRINEC) were calculated [13]. Site of NSTI was noted (cephalic, trunk, pelvis, and limbs), the proportion of injured skin surface was assessed according to the nine rules for burns [14]. Clinical and therapeutic features were recorded on admission and during the ICU stay, including septic shock, need for mechanical ventilation, vasoactive support, renal failure, and renal replacement therapy. The lengths of ICU and hospital stay and the day-28, day-90, and 1-year mortality rates after ICU admission were collected.

### Surgical procedures and microbiological data

To confirm the diagnosis and to achieve source control through aggressive debridement of infected necrotic tissue, a surgical exploration was performed. Surgical samples obtained from deep tissues collected during the initial surgical source control were obtained for microbiological analysis. Swabs and samples from nonsterile sites (such as open bullae) were not considered. Wound care, including cleaning and trimming, was performed every 24 or 48 h at bedside according to the local aspect. In case of unfavorable systemic and/or local evolution, a second surgical exploration was performed in the operating room. Blood cultures were collected on the day of source control surgery and the next 3 days.

Microbiological samples were sent to the laboratory for bacterial and fungal cultures and were processed according to standard laboratory methods. Plates were incubated for 48 h at 35 °C. All morphologically distinct colonies were identified by standard techniques and tested for antibiotic susceptibility by the disk diffusion method according to EUCAST [15]. Multidrug resistance (MDR) was defined according to international definitions [16].

### Antimicrobial therapy

Empirical anti-infective therapy, taking into account clinical severity, was initiated and targeted Gram-positive and Gram-negative aerobic and anaerobic microorganisms. Our analyses compared patients who received antimicrobial therapy ≥ 24 h before the first NSTI sample collected (called the exposed group) versus patients free of antibiotics before samples (called the unexposed group).

The conventional empirical regimen was based on initial severity and used beta-lactams with anti-anaerobic activity (amoxicillin-clavulanate, piperacillin/tazobactam, or imipenem/cilastatin) or third-generation cephalosporin associated with metronidazole combined ± aminoglycosides and ± anti-Gram-positive agents in case of suspicion of resistant bacteria [2, 17]. A specific analysis was performed for those receiving empirical piperacillin-tazobactam or carbapenem therapy. Documented anti-infective therapy was adapted to the results of identification and susceptibility testing (≥ 48 h) and was defined as adequate when targeting all the cultured microorganisms. Antimicrobial de-escalation was defined as the discontinuation of one or more components of combination empirical therapy, and/or the change from a broad-spectrum to a narrower spectrum antimicrobial was then performed as soon as possible [18].

Adjuvant therapies such as hyperbaric oxygenation therapy or intravenous immunoglobulin infusion were not performed in our center.

### Statistical analysis

The results are expressed as medians and IQRs and as absolute numbers and proportions for categorical data. Continuous data were compared using the Mann–Whitney *U* test. Categorical data were analyzed using the Chi2 or Fisher’s exact test. All reported statistical tests were 2-sided, and *p* values < 0.05 were considered significant. Statistical analyses were performed using R software version 3.6.2.

The study was organized in agreement with the STROBE recommendations.

## Results

### Study population

Overall, 100 patients admitted to our ICU for severe NSTI were analyzed. For 23 (23%) patients, antimicrobial therapy was started ≥ 24 h before surgical sampling (exposed group), and 77 (77%) patients belonged to the unexposed group. Patient characteristics, NSTI sites and severity scores are presented in Table 1. No differences were found between the two groups.

**Table 1:** Baseline features of the overall study patients and the antibiotic-exposed and unexposed groups

	Overall *n* = 100	Unexposed group *n* = 77	Exposed group *n* = 23	*p* value
Age (years), median [IQR]	58 [50–68]	59 [50–68]	57 [52–61]	0.371
Male sex, *n* (%)	63 (63)	47 (61)	16 (70)	0.623
BMI (kg/m^2^), median [IQR]	28 [23–34]	27 [22–32]	30 [26–35]	0.102
Localization: limbs, *n* (%)	52 (52)	38 (49)	14 (61)	0.353
Localization: pelvis, *n* (%)	15 (15)	11 (14)	4 (17)	0.743
Localization: cephalic, *n* (%)	23 (23)	21 (27)	2 (9)	0.09
Localization: trunk, *n* (%)	13 (13)	8 (10)	5 (22)	0.169
Skin surface (%), median [IQR]	4.5 [4.5–9]	4.5 [4.5–9]	4.5 [4.5–9]	0.597
SAPS-II score on admission, median [IQR]	28 [23–37]	28 [23–37]	25 [21–36]	0.287
SOFA score on admission, median [IQR]	5 [3-6]	5 [3-6]	4 [3-7]	0.977
LRINEC score on admission, median [IQR]	2 [1-4]	2 [0–4]	3 [2-5]	0.118
Septic shock on admission, n (%)	65 (65)	51 (66)	14 (61)	0.628
Charlson score on admission, median [IQR]	4 [3-7]	3 [2-6]	3 [2-6]	0.758
Underlying medical conditions				
Malignancy, *n* (%)	23 (23)	20 (26)	3 (13)	0.264
Obesity, *n* (%)	24 (23)	16 (21)	7 (30)	0.399
Diabetes mellitus, *n* (%)	24 (24)	17 (22)	7 (30)	0.415
Coronary disease, *n* (%)	20 (20)	14 (18)	6 (26)	0.553
Peripheral vascular disease, *n* (%)	17 (17)	13 (17)	4 (17)	1
Alcohol use, *n* (%)	20 (20)	16 (21)	4 (17)	1
Active smoking, *n* (%)	54 (54)	42 (55)	12 (52)	1
Immunosuppression, *n* (%)	10 (10)	8 (10)	2 (9)	1

*BMI* body mass index, *LRINEC* Laboratory Risk Indicators for Necrotizing Fasciitis score, *SAPS*-*II* Simplified Acute Physiology Score-II, *SOFA* sequential organ failure assessment

### Antimicrobial therapy and bacterial species

In the exposed group, the early initiation of anti-infective was decided by the attending physician before surgery and ICU admission (general practitioners in 2 cases and physicians in charge in the medical or surgical ward in 21 cases, respectively). The underlying diseases (diabetes mellitus, obesity) and the anatomic location (trunk, limb, and pelvis) were the key drivers of the early prescription (Table 1). In the exposed group, the median duration of antimicrobial therapy before operative sampling was 6 days. Overall, microbiological cultures of surgical samples were positive in 84 patients and negative in 16 patients, including 3/23 (13%) patients in the exposed group and 13/77 (17%) patients in the unexposed group (*p* = 0.70). The distribution of microorganisms is presented in Table 2. No differences were observed between the two groups. Polymicrobial samples were found in 38 (49.4%) patients in the unexposed group and 11 (47.8%) patients in the exposed group (*p* = 1.0). Bacteremia was reported in 24% of the population, with the same bacterial species as in the operative samples, and there was no significant difference between the two groups (exposed group, *n* = 19 (26%) vs. unexposed group, *n* = 5 (24%), *p* = 1.0).

**Table 2:** Distribution of microorganisms at the time of the first sampling in the groups exposed and unexposed to antibiotics

	Unexposed group	Exposed group	*p* value
Aerobes, *n* (%)	**111 (86)**	**31 (76)**	**0.326**
Gram-positive bacteria, *n* (%)	**71 (55)**	**16 (39)**	**0.207**
Enterococci, *n* (%)	12 (9)	4 (10)	1
Streptococci, *n* (%)	37 (29)	8 (20)	0.341
GAS, *n* (%)	10 (8)	2 (5)	0.728
Staphylococci, *n* (%)	24 (19)	4 (10)	0.419
*Staphylococcus aureus*, *n* (%)	12 (9)	3 (7)	1
Coagulase-negative staphylococci, *n* (%)	10 (8)	1 (2)	0.449
Gram-negative bacteria, *n* (%)	**40 (31)**	**15 (37)**	**0.476**
*Enterobacterales*, *n* (%)	34 (26)	12 (29)	0.634
*Escherichia coli*, *n* (%)	15 (12)	6 (15)	0.562
Nonfermenting Gram-negative bacilli, *n* (%)	6 (5)	3 (7)	0.426
*Pseudomonas aeruginosa*, *n* (%)	6 (5)	3 (7)	0.426
Anaerobes, *n* (%)	**15 (12)**	**7 (23)**	**0.267**
Fungi, *n* (%)	**3 (2)**	**3 (7)**	**0.133**
Total number of strains, *n* (%)	**129**	**41**	
Total number of MDR bacteria,* n* (%)	**7 (5)**	**1 (2)**	**1**
MRSA, *n* (%)	2 (3)	0 (0)	1
Overproduction of intrinsic or plasmid-encoded AmpC, *n* (%)	2 (3)	0 (0)	1
ESBL producing *Enterobacterales*, *n* (%)	2 (3)	1 (2)	0.548
Carbapenemase producing *Enterobacterales*, *n* (%)	1 (1)	0 (0)	1

*ESBL* extended spectrum beta-lactamase, *MDR* multidrug resistance, *MRSA* methicillin-resistant *Staphylococcus aureus*

The main antimicrobial regimens for empirical therapy were similar between the two groups (Table 3). The proportions of adequacy were comparable between the two groups (*n* = 60 (84.5%) in the unexposed group vs. *n* = 19 (86.4%) in the exposed group, *p* = 0.482). The use of amoxicillin-clavulanate was not significantly different between the two groups (22 (29%) vs. 3 (13%), *p* = 0.174), while piperacillin/tazobactam or carbapenems were more frequently administered in the exposed group than in the unexposed group (15/23 (65%) vs. 28/77 (36%), respectively *p* = 0.0175). Interestingly, the severity scores were similar in patients receiving piperacillin/tazobactam or carbapenems vs. the other patients (SAPS-II: 27 [22–36] vs. 28 [23–38], *p* = 0.542; SOFA score: 4 [3-6] vs. 5 [4-7], *p* = 0.085). Moreover, in the specific group of patients exposed to antimicrobial therapy, no difference was found between patients receiving these regimens and the other patients (SAPS-II: 25 [21–37] vs. 23 [21–32], *p* = 0.332; SOFA score: 4 [3-6] vs. 6 [4-8], *p* = 0.104). In the nonexposed group, no differences were found between patients receiving piperacillin/tazobactam or carbapenems vs. the other patients (SAPS-II: 28 [23–38] vs. 28 [23–36], *p* = 0.571; SOFA score: 4 [3-6] vs. 5 [3-6], *p* = 0.321).

**Table 3:** Antimicrobial therapy

First-line antimicrobial therapy	Unexposed group *n* = 77	Exposed group *n* = 23	*p* value
Amoxicillin-clavulanate, *n* (%)	22 (29)	3 (13)	0.174
Piperacillin-tazobactam, *n* (%)	22 (29)	11 (48)	0.128
Carbapenems, *n* (%)	6 (8)	4 (17)	0.232
Cephalosporins, *n* (%)	16 (21)	2 (9)	0.231
Metronidazole, *n* (%)	15 (20)	2 (9)	0.346
Aminoglycosides, *n* (%)	18 (23)	6 (26)	0.786
Adequate antimicrobial therapy, *n* (%)	70 (91)	21 (91)	1
Therapeutic de-escalation, *n* (%)	43 (60)	14 (64)	0.807
Nondocumented NSTI, *n* (%)	11 (15)	2 (9)	0.794

*NSTI* necrotizing soft-tissue infections

Therapeutic de-escalation was performed in 42 (59%) patients in the unexposed group and in 15 (65%) patients in the exposed group (*p* = 0.634). The proportions of de-escalation were similar in patients receiving piperacillin/tazobactam or carbapenems versus the other patients (26 (65%) vs. 31 (57%), *p* = 0.525). Interestingly, in the exposed group, de-escalation was performed in 10 (71%) patients who received piperacillin/tazobactam or carbapenems and in 4 (50%) patients who did not receive these regimens.

The median duration of antimicrobial therapy from the index surgery was 15 days in the exposed group and 14 days in the unexposed group (*p* = 0.202). In the subgroup of patients with negative microbiological cultures, the median durations of antimicrobial therapy were 14 days in the exposed group and 12 days in the unexposed group (*p* = 1.0).

### Outcome

No statistically significant differences were found between the two groups regarding ICU and hospital lengths of stay and mortality rates (Table 4). In univariate analysis, the 1-year mortality rate was stable throughout the inclusion period among patients with NSTI (*p* = 0.786). De-escalation was associated with significantly less mortality at day 28 (14 (38) vs. 10 (18), *p* = 0.032) but was not associated with different antibiotic durations (same median of 15 days, *p* = 0.929) or different hospital lengths of stay (*p* = 0.869).

**Table 4:** Outcomes

	Unexposed group *n* = 77	Exposed group *n* = 23	*p* value
Supportive therapy during the ICU stay			
Renal replacement therapy, *n* (%)	15 (21)	7 (30)	0.395
Need of reoperation, *n* (%)	38 (49)	11 (48)	0.741
Number of bedside debridement, median [IQR]	4 [1, 10]	6 [2, 10]	0.446
Length of mechanical ventilation, median [IQR]	3 [0, 12]	9 [0, 14]	0.596
Length of hospital stay in days, median [IQR]	39 [18, 59]	47 [40, 60]	0.208
Length of ICU stay in days, median [IQR]	7 [2, 19]	11 [4, 20]	0.446
Mortality at day 28, *n* (%)	14 (18)	4 (17)	1
Mortality at day 90, *n* (%)	19 (25)	4 (17)	0.578
Mortality at 1 year, *n* (%)	20 (26)	4 (17)	0.579

*ICU* intensive care unit

The mortality rates at 1 year were similar between patients with negative microbiological cultures and patients with positive microbiological cultures (*n* = 3 (23%) vs. *n* = 22 (25%), *p* = 1.0). Similarly, the mortality rates at 1 year were similar in patients receiving piperacillin/tazobactam or carbapenems vs. the other patients (*n* = 9 (21%) vs. *n* = 16 (28%), *p* = 0.488).

## Discussion

In this retrospective cohort of 100 consecutive patients admitted to the ICU for severe NSTI, we observed that antimicrobial therapy given more than 24 h before surgical sampling does not sterilize microbiological cultures and seems to have only a minimal impact on microbiological findings and patient outcome. In addition, our data suggest that previous antimicrobial therapy selection pressure should not be an incentive for prescribing broad-spectrum therapy.

The issue of previous antibiotic administration before microbiological sampling varies largely from 24.5 to 61.2% of patients with skin and soft tissue infections [19, 20]. To the best of our knowledge, our study is the first to focus on the role of previous antimicrobial therapy administration in critically ill patients with NSTI. This question highlights two conflicting issues. The need for microbiological samples is highly relevant for an accurate diagnosis and adequate antimicrobial therapy [5, 7]. However, appropriate antimicrobial therapy should be initiated as quickly as possible, taking into account the clinical severity [5].

A recent study emphasized the importance of delayed appropriate therapy in patients with severe bacterial infection [21]. In this meta-analysis involving more than 9000 patients, the mortality rate was significantly decreased in patients receiving timely appropriate therapy compared with patients receiving delayed antibiotic therapy (OR 0.57; 95% CI, 0.45–0.72). Interestingly, there was no significant difference in time to appropriate therapy (from 3.8 to 166 h) between those who died and the survivors. However, in this cohort of severe bacterial infections, the proportion of severe NSTI patients was very limited.

The importance of the initiation of antimicrobial therapy before or after the collection of microbiological samples is an old debate. In many reports, the proportion of sterilized samples appears to be higher in patients receiving antimicrobial therapy before surgical or perioperative sampling. In a study involving 163 septic patients from an emergency department, the authors observed that cultures were more frequently positive in patients who received antimicrobial therapy after the collection of microbiological samples than in those receiving antimicrobial therapy before sampling (80.4% vs. 46.7%, *p* < 0.005) [22]. A recent large retrospective study involving 27,792 non-ICU patients confirmed these observations [23]. Interestingly, in this study, there were some differences depending on the source of the infection: the proportions of positive blood and urine specimens taken before antimicrobial therapy were higher than those taken after sampling (21.3% vs. 15.1 and 21.7% vs. 12.0%, respectively, both *p* < 0.001), while the proportion of other positive samples (including respiratory secretions and body fluid specimens) was lower in patients receiving antimicrobial therapy before sampling than in those receiving therapy after sampling [23]. Obviously, sterilizing samples at deep surgical sites such as in cases of meningitis, endocarditis, or infected pancreatitis seems to be more difficult to achieve [24], while the deleterious effects of previous antibiotic therapy before microbiological samples have been stressed in several conditions, such as meningitis or endocarditis. Extrapolation to SSTIs is difficult, as data related to the tissue diffusion of antibiotics are scarce. However, we can assume adequate diffusion based on the clinical response, as mentioned by Bruun et al., who reported a cessation of lesion spread and improvement of local inflammation within 3 days in 90% of a cohort of 216 patients with cellulitis [19].

Another source of concern is the susceptibility of bacterial species following a previous antibiotic therapy. In our study, we did not observe any difference in bacterial species or in the susceptibility profile between the two groups, including the proportion of MDR bacteria. Several studies addressed this issue, all of them out of the field of NSTI. In a study by Garret et al. involving 137 patients with necrotizing pancreatitis, the proportion of patients developing an infection from pancreatic samples with MDR bacteria was similar in the exposed and unexposed groups (24 and 26%, respectively; *p* = 0.99) [25]. The results of other studies are more controversial. Montravers et al. observed that starting antibiotic therapy before sample collection was associated with less-susceptible microorganisms in a population of patients with nosocomial pneumonia [26]. Interestingly, Li et al. underlined a difference in pathogens between exposed and unexposed patients before sampling, especially in case of bacteremia or urine samples [23]. In addition, no difference was found in the case of body fluid specimens. However, it is difficult to extrapolate strong conclusions to NSTIs from these data.

In line with previous studies, our results allow us to address several important issues. First, in these life-threatening infections, the initiation of empirical therapy before surgical sampling seems to be a safe approach that does not jeopardize the quality of the microbiological samples. Nevertheless, this fundamental observation raises questions about the role of antibiotic therapy in relation to surgical management, a key factor in terms of prognosis.

Second, the absence of differences in terms of ecology or emergence of resistance in the context of severe NSTI depending on the delay of initiation of antimicrobial therapy adds complexity to the consideration of the benefit-risk balance of administering empirical therapy prior to surgical sampling.

Third, our results inevitably raise the question of the persistence of pathogens within surgical specimens despite prolonged antimicrobial therapy in the exposed group. This remark highlights the lack of studies on antimicrobial therapy pharmacokinetics in the context of NSTI [27, 28]. Most of the data addressing the issue of drug penetration into skin and soft tissue have been collected from healthy volunteers through skin blister or microdialysis techniques and from patients treated for diabetic foot infection [27]. These studies have demonstrated reduced tissue diffusion of hydrophobic antimicrobial agents versus lipophilic drugs in patients with SSTI, with a specific relevance for diabetic patients. However, even in diabetic patients, it seems difficult to extrapolate the results related to microangiopathy to what is observed in NSTI. Nevertheless, the combined adequacy of source control and antimicrobials constitutes a major component of improved management [29].

Based on our observations, the large empirical use of piperacillin/tazobactam and carbapenems in the exposed group representing 2/3 of the prescriptions should be questioned. Compared to the other patients, those receiving these broad-spectrum regimens had a similar initial severity score, underwent a higher rate of de-escalation with a similar mortality rate at 1 year. The use of rapid diagnostic test and early identification of resistant microorganisms could limit this selection pressure in a near future. Local policies vary largely for the selection of empirical therapy. For instance, Madsen et al. reported a massive use of meropenem (85% of all antibiotics) in their cohort of NSTI cases, a proportion rarely achieved in other series. In a previous work, we compared the microbiology of two surgical procedures in the context of NSTI patients, and we showed that the selection of MDR bacteria occurs later during the management of these patients, which seems consistent with these present results [4].

Nevertheless, our results should be considered cautiously given several limitations. This retrospective study conducted over a prolonged period of time involved only 100 patients. However, the 1-year mortality was stable throughout the inclusion period. In addition, the heterogeneity of the cohort (limb, pelvis, cephalic, trunk) could be a source of bias. We chose a 24-h cutoff, as reported by some other authors [25], because it seemed to be a good reflection of clinical rationality. Nevertheless, other more restrictive cutoffs could have been used. Moreover, the retrospective nature of this study does not allow us to clearly demonstrate the reasons for the early initiation of antibiotic therapy in some patients. Nonetheless, diabetes mellitus, obesity, and trunk or pelvic NSTIs appear to be incentive features for early antibiotic administration. A prospective analysis could bring further information. Lastly, because of the retrospective and exploratory nature of the study, we did not perform any power calculation [30].

## Conclusion

Our results suggest that prior antibiotic therapy in the context of severe ICU NSTI does not interfere with any sterilization of the specimens and is unrelated to the microbiology and patient outcome. Moreover, contrary to recent international recommendations emphasizing the role of broad-spectrum antibiotic therapy targeting MDR Gram-negative microorganisms [2], this policy does not seem to be necessary in view of our local microbiological data. These results are a strong incentive for tailoring empirical anti-infective therapy to the local epidemiology. Prospective multicenter studies are required to confirm our observations.

## Data Availability

The datasets used and analyzed in this study are available from the corresponding author upon reasonable request.
